# *cis*-Diaqua­bis­(2,2′-bipyrimidine-κ^2^ *N* ^1^,*N* ^1′^)manganese(II) bis­(perchlorate) nitro­methane disolvate monohydrate

**DOI:** 10.1107/S1600536811045612

**Published:** 2011-11-05

**Authors:** Kwang Ha

**Affiliations:** a School of Applied Chemical Engineering, The Research Institute of Catalysis, Chonnam National University, Gwangju 500-757, Republic of Korea

## Abstract

The asymmetric unit of the title compound, [Mn(C_8_H_6_N_4_)_2_(H_2_O)_2_](ClO_4_)_2_·2CH_3_NO_2_·H_2_O, contains one half of a cationic Mn^II^ complex, a ClO_4_^−^ anion, a nitro­methane solvent mol­ecule and one half-mol­ecule of water. The complex mol­ecule and the solvent water mol­ecule are located on a twofold rotation axis. In the complex, the Mn^II^ ion has a distorted *cis*-N_4_O_2_ octa­hedral coordination geometry defined by four N atoms of the two chelating 2,2′-bipyrimidine ligands and two O atoms of water mol­ecules. In the crystal, the complex cations, anions and solvent mol­ecules are linked by inter­molecular O—H⋯N, O—H⋯O and weak C—H⋯O hydrogen bonds. The ClO_4_^−^ anion is disordered over two sites with a site-occupancy factor of 0.512 (12) for the major component.

## Related literature

For related structures of 2,2′-bipyrimidine Mn^II^ complexes, see: Hong *et al.* (1996 ▶); Ha (2011 ▶).
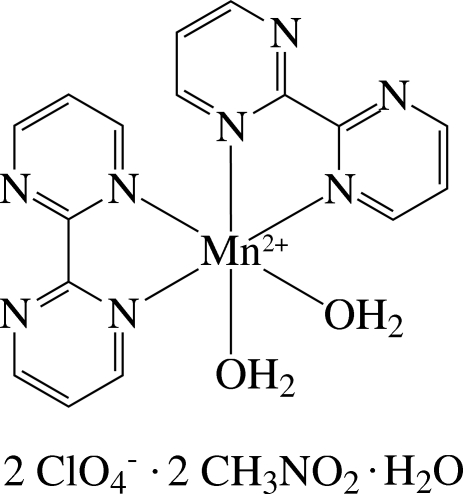


## Experimental

### 

#### Crystal data

[Mn(C_8_H_6_N_4_)_2_(H_2_O)_2_](ClO_4_)_2_·2CH_3_NO_2_·H_2_O
*M* *_r_* = 746.31
Monoclinic, 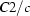

*a* = 21.913 (3) Å
*b* = 9.1956 (14) Å
*c* = 15.106 (2) Å
β = 101.756 (3)°
*V* = 2980.1 (8) Å^3^
*Z* = 4
Mo *K*α radiation
μ = 0.71 mm^−1^
*T* = 200 K
0.35 × 0.27 × 0.24 mm

#### Data collection

Bruker SMART 1000 CCD diffractometer
Absorption correction: multi-scan (*SADABS*; Bruker, 2000 ▶) *T*_min_ = 0.830, *T*_max_ = 1.000
10552 measured reflections
3617 independent reflections
2302 reflections with *I* > 2σ(*I*)
*R*_int_ = 0.044

#### Refinement

*R*[*F* ^2^ > 2σ(*F* ^2^)] = 0.059
*wR*(*F* ^2^) = 0.199
*S* = 1.06
3617 reflections
208 parameters
H-atom parameters constrained
Δρ_max_ = 1.05 e Å^−3^
Δρ_min_ = −0.69 e Å^−3^

### 

Data collection: *SMART* (Bruker, 2000 ▶); cell refinement: *SAINT* (Bruker, 2000 ▶); data reduction: *SAINT*; program(s) used to solve structure: *SHELXS97* (Sheldrick, 2008 ▶); program(s) used to refine structure: *SHELXL97* (Sheldrick, 2008 ▶); molecular graphics: *ORTEP-3* (Farrugia, 1997 ▶) and *PLATON* (Spek, 2009 ▶); software used to prepare material for publication: *SHELXL97*.

## Supplementary Material

Crystal structure: contains datablock(s) global, I. DOI: 10.1107/S1600536811045612/xu5372sup1.cif

Structure factors: contains datablock(s) I. DOI: 10.1107/S1600536811045612/xu5372Isup2.hkl

Additional supplementary materials:  crystallographic information; 3D view; checkCIF report

## Figures and Tables

**Table 1:** Selected bond lengths (Å)

Mn1—O1	2.176 (2)
Mn1—N1	2.258 (3)
Mn1—N4	2.249 (3)

**Table 2:** Hydrogen-bond geometry (Å, °)

*D*—H⋯*A*	*D*—H	H⋯*A*	*D*⋯*A*	*D*—H⋯*A*
O1—H1*A*⋯N2^i^	0.84	2.48	3.286 (4)	162
O1—H1*A*⋯N3^i^	0.84	2.29	2.879 (4)	128
O1—H1*B*⋯O8^ii^	0.84	1.97	2.757 (4)	156
O8—H8*A*⋯O2^iii^	0.84	1.95	2.792 (4)	175
C3—H3⋯O6^iv^	0.95	2.54	3.346 (5)	143
C6—H6⋯O3*A*^v^	0.95	2.58	3.239 (7)	127
C8—H8⋯O6^vi^	0.95	2.59	3.175 (5)	120
C9—H9*C*⋯O5*A*	0.98	2.52	3.388 (9)	148

Symmetry codes: (i) 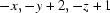; (ii) 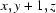; (iii) 
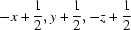
; (iv) 
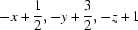
; (v) 
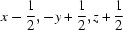
; (vi) 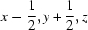.

**Fig. 1.**
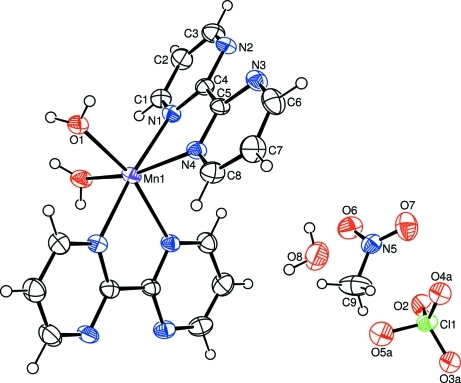
The structure of the title compound, with displacement ellipsoids drawn at the 40% probability level for non-H atoms. Unlabelled atoms are related to the reference atoms by the (-x, y, 1/2 - z) symmetry transformation. For the sake of clarity, only the major disorder component is shown.

**Fig. 2.**
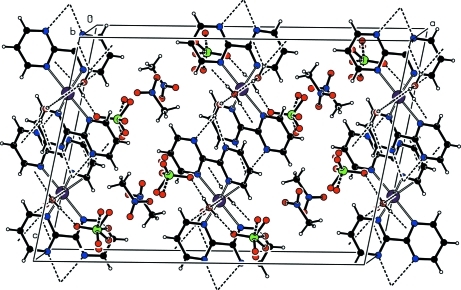
View of the unit-cell contents of the title compound. Hydrogen-bond interactions and the bonds of the disordered anions are drawn with dashed lines.

## supplementary crystallographic information

### Comment

Mononuclear Mn^II^ complexes of 2,2'-bipyrimidine (bpym; C_8_H_6_N_4_) ligand and ClO_4_^-^ anions, such as [Mn(bpym)_2_(H_2_O)_2_](ClO_4_)_2_.2H_2_O (Hong *et al.*, 1996) and [Mn(bpym)_2_(CH_3_CN)(H_2_O)][Mn(bpym)_2_(H_2_O)_2_](ClO_4_)_4_.2H_2_O (Ha, 2011), have been investigated previously.

The asymmetric unit of the title compound, [Mn(bpym)_2_(H_2_O)_2_](ClO_4_)_2_.2CH_3_NO_2_.H_2_O, contains one half of a cationic Mn^II^ complex, a ClO_4_ anion, a nitromethane solvent molecule and one half of a water molecule (Fig. 1). The complex and the solvent water molecule are located on the twofold rotation axis running in the [010] direction and passing through the atoms Mn1 and O8. In the complex, the Mn^II^ ion has a distorted *cis*-N_4_O_2_ octahedral coordination geometry defined by four N atoms of the two chelating bpym ligands and two O atoms of water ligands. The tight N—Mn—N chelating angles contribute the distortion of the ocataheron [<N1—Mn1—N4 = 72.59 (10)°], which result in non-linear *trans* axes [<O1—Mn1—N4^i^ = 161.83 (10)° and <N1—Mn1—N1^i^ = 174.05 (14)°); symmetry code i: -*x*,*y*,1/2 - *z*]. The Mn—N bond lengths are almost equivalent and slightly longer than the Mn—O bond (Table 1). The dihedral angle between the least-squares planes of the two bpym ligands [maximum deviation = 0.087 (3) Å] is 75.44 (4)°. In the crystal structure, the complex molecules, anions and solvent molecules are linked by intermolecular O—H···N, O—H···O and C—H···O hydrogen bonds (Fig. 2, Table 2). The complexes are stacked in columns along the *b* axis. When viewed down the *c* axis, the successive complexes stack in the opposite manner. In the columns, several intermolecular π-π interactions between adjacent pyrimidine rings are present, the shortest ring centroid-centroid distance being 3.688 (2) Å.

### Experimental

To a solution of Mn(ClO_4_)_2_.6H_2_O (0.3617 g, 0.999 mmol) in EtOH (20 ml) was added 2,2'-bipyrimidine (0.1586 g, 1.003 mmol) and stirred for 3 h at room temperature. The formed precipitate was separated by filtration, washed with EtOH and dried at 50°C, to give a pale yellow powder (0.2713 g). Crystals suitable for X-ray analysis were obtained by slow evaporation from a CH_3_NO_2_ solution.

### Refinement

Carbon-bound H atoms were positioned geometrically and allowed to ride on their respective parent atoms [C—H = 0.95 Å (CH) or 0.98 Å (CH_3_) and *U*_iso_(H) = 1.2*U*_eq_(C) or 1.5*U*_eq_(methyl C)]. Oxygen-bound H atoms were located from Fourier difference maps then allowed to ride on their parent O atoms in the final cycles of refinement with O—H = 0.84 Å and *U*_iso_(H) = 1.5 *U*_eq_(O). The ClO_4_^-^ anion displayed relatively large displacement factors and low electron density peaks so that the anion appears to be highly disordered. Atoms O3, O4 and O5 were modelled isotropically as disordered over two sites with a site occupancy factor of 0.51 (1) for the major component. The highest peak (1.05 e Å^-3^) and the deepest hole (-0.69 e Å^-3^) in the difference Fourier map are located 0.81 Å and 0.66 Å from the atoms O3A and O5B, respectively.

### Crystal data

**Table:**

[Mn(C_8_H_6_N_4_)_2_(H_2_O)_2_](ClO_4_)_2_·2CH_3_NO_2_·H_2_O	*F*(000) = 1524
*M**_r_* = 746.31	*D*_x_ = 1.663 Mg m^−^^3^
Monoclinic, *C*2/*c*	Mo *K*α radiation, λ = 0.71073 Å
Hall symbol: -C 2yc	Cell parameters from 3688 reflections
*a* = 21.913 (3) Å	θ = 2.4–28.2°
*b* = 9.1956 (14) Å	µ = 0.71 mm^−^^1^
*c* = 15.106 (2) Å	*T* = 200 K
β = 101.756 (3)°	Stick, pale yellow
*V* = 2980.1 (8) Å^3^	0.35 × 0.27 × 0.24 mm
*Z* = 4	

### Data collection

**Table:**

Bruker SMART 1000 CCD diffractometer	3617 independent reflections
Radiation source: fine-focus sealed tube	2302 reflections with *I* > 2σ(*I*)
graphite	*R*_int_ = 0.044
φ and ω scans	θ_max_ = 28.3°, θ_min_ = 1.9°
Absorption correction: multi-scan (*SADABS*; Bruker, 2000)	*h* = −29→29
*T*_min_ = 0.830, *T*_max_ = 1.000	*k* = −12→11
10552 measured reflections	*l* = −19→20

### Refinement

**Table:**

Refinement on *F*^2^	Primary atom site location: structure-invariant direct methods
Least-squares matrix: full	Secondary atom site location: difference Fourier map
*R*[*F*^2^ > 2σ(*F*^2^)] = 0.059	Hydrogen site location: inferred from neighbouring sites
*wR*(*F*^2^) = 0.199	H-atom parameters constrained
*S* = 1.06	*w* = 1/[σ^2^(*F*_o_^2^) + (0.1146*P*)^2^] where *P* = (*F*_o_^2^ + 2*F*_c_^2^)/3
3617 reflections	(Δ/σ)_max_ = 0.001
208 parameters	Δρ_max_ = 1.05 e Å^−^^3^
0 restraints	Δρ_min_ = −0.69 e Å^−^^3^

### Special details

**Table:**

Geometry. All e.s.d.'s (except the e.s.d. in the dihedral angle between two l.s. planes) are estimated using the full covariance matrix. The cell e.s.d.'s are taken into account individually in the estimation of e.s.d.'s in distances, angles and torsion angles; correlations between e.s.d.'s in cell parameters are only used when they are defined by crystal symmetry. An approximate (isotropic) treatment of cell e.s.d.'s is used for estimating e.s.d.'s involving l.s. planes.
Refinement. Refinement of *F*^2^ against ALL reflections. The weighted *R*-factor *wR* and goodness of fit *S* are based on *F*^2^, conventional *R*-factors *R* are based on *F*, with *F* set to zero for negative *F*^2^. The threshold expression of *F*^2^ > σ(*F*^2^) is used only for calculating *R*-factors(gt) *etc*. and is not relevant to the choice of reflections for refinement. *R*-factors based on *F*^2^ are statistically about twice as large as those based on *F*, and *R*- factors based on ALL data will be even larger.

### Fractional atomic coordinates and isotropic or equivalent isotropic displacement parameters (Å^2^)

**Table:**

	*x*	*y*	*z*	*U*_iso_*/*U*_eq_	Occ. (<1)
Mn1	0.0000	1.01984 (8)	0.2500	0.0309 (2)	
O1	−0.04920 (12)	1.1867 (3)	0.31005 (16)	0.0423 (6)	
H1A	−0.0587	1.1700	0.3602	0.063*	
H1B	−0.0409	1.2738	0.3007	0.063*	
N1	0.08152 (12)	1.0071 (3)	0.36833 (18)	0.0303 (6)	
N2	0.10990 (15)	0.9334 (3)	0.52207 (18)	0.0391 (7)	
N3	0.00248 (13)	0.7724 (3)	0.49924 (18)	0.0380 (7)	
N4	−0.02402 (13)	0.8545 (3)	0.34722 (17)	0.0319 (6)	
C1	0.13597 (16)	1.0798 (4)	0.3762 (2)	0.0375 (8)	
H1	0.1446	1.1316	0.3258	0.045*	
C2	0.17891 (18)	1.0796 (4)	0.4560 (3)	0.0445 (9)	
H2	0.2177	1.1284	0.4615	0.053*	
C3	0.16387 (19)	1.0065 (4)	0.5278 (3)	0.0475 (9)	
H3	0.1928	1.0074	0.5840	0.057*	
C4	0.07234 (15)	0.9357 (3)	0.4413 (2)	0.0306 (7)	
C5	0.01357 (15)	0.8495 (4)	0.42920 (19)	0.0305 (7)	
C6	−0.04833 (19)	0.6911 (4)	0.4843 (3)	0.0478 (9)	
H6	−0.0573	0.6347	0.5328	0.057*	
C7	−0.08852 (19)	0.6844 (4)	0.4026 (3)	0.0507 (10)	
H7	−0.1242	0.6232	0.3931	0.061*	
C8	−0.07495 (17)	0.7708 (4)	0.3339 (3)	0.0414 (8)	
H8	−0.1023	0.7706	0.2764	0.050*	
Cl1	0.35218 (4)	0.06501 (11)	0.11241 (6)	0.0450 (3)	
O2	0.41653 (14)	0.0994 (4)	0.1248 (2)	0.0629 (9)	
O3A	0.3343 (3)	−0.0585 (8)	0.0577 (5)	0.055 (2)*	0.512 (12)
O4A	0.3261 (3)	0.0650 (8)	0.1904 (4)	0.059 (2)*	0.512 (12)
O5A	0.3161 (3)	0.1896 (8)	0.0597 (6)	0.073 (2)*	0.512 (12)
O3B	0.3318 (4)	−0.0053 (11)	0.0305 (7)	0.080 (3)*	0.488 (12)
O4B	0.3488 (5)	−0.0100 (12)	0.1960 (7)	0.099 (3)*	0.488 (12)
O5B	0.3249 (5)	0.2003 (12)	0.1121 (9)	0.107 (4)*	0.488 (12)
O6	0.29523 (16)	0.4196 (3)	0.2512 (2)	0.0631 (9)	
O7	0.25300 (16)	0.2492 (4)	0.3104 (2)	0.0721 (10)	
N5	0.25476 (15)	0.3308 (3)	0.2479 (2)	0.0434 (7)	
C9	0.2066 (2)	0.3224 (7)	0.1673 (3)	0.0796 (16)	
H9A	0.1940	0.4209	0.1464	0.119*	
H9B	0.1706	0.2702	0.1808	0.119*	
H9C	0.2225	0.2705	0.1201	0.119*	
O8	0.0000	0.4351 (4)	0.2500	0.0667 (13)	
H8A	0.0238	0.4889	0.2866	0.100*	

### Atomic displacement parameters (Å^2^)

**Table:**

	*U*^11^	*U*^22^	*U*^33^	*U*^12^	*U*^13^	*U*^23^
Mn1	0.0335 (4)	0.0380 (4)	0.0215 (4)	0.000	0.0063 (3)	0.000
O1	0.0559 (16)	0.0434 (14)	0.0278 (12)	0.0113 (12)	0.0088 (11)	−0.0014 (10)
N1	0.0273 (14)	0.0367 (15)	0.0272 (14)	0.0031 (11)	0.0065 (11)	−0.0004 (10)
N2	0.0457 (18)	0.0441 (17)	0.0240 (14)	0.0028 (13)	−0.0013 (13)	−0.0013 (12)
N3	0.0424 (17)	0.0447 (17)	0.0299 (15)	0.0020 (13)	0.0143 (13)	0.0051 (12)
N4	0.0356 (15)	0.0363 (15)	0.0253 (13)	0.0012 (11)	0.0099 (11)	−0.0006 (11)
C1	0.0350 (19)	0.042 (2)	0.0363 (18)	−0.0022 (14)	0.0091 (15)	−0.0024 (14)
C2	0.033 (2)	0.049 (2)	0.048 (2)	−0.0020 (15)	0.0001 (17)	−0.0024 (17)
C3	0.045 (2)	0.054 (2)	0.038 (2)	−0.0024 (17)	−0.0055 (17)	−0.0064 (17)
C4	0.0337 (17)	0.0338 (17)	0.0261 (16)	0.0058 (13)	0.0101 (13)	−0.0031 (12)
C5	0.0358 (18)	0.0358 (17)	0.0219 (15)	0.0020 (13)	0.0106 (13)	−0.0016 (12)
C6	0.052 (2)	0.054 (2)	0.042 (2)	−0.0003 (18)	0.0208 (19)	0.0139 (17)
C7	0.049 (2)	0.050 (2)	0.056 (2)	−0.0115 (18)	0.017 (2)	0.0038 (19)
C8	0.038 (2)	0.047 (2)	0.0386 (19)	−0.0077 (15)	0.0080 (16)	−0.0030 (15)
Cl1	0.0442 (6)	0.0512 (6)	0.0427 (5)	−0.0063 (4)	0.0164 (4)	−0.0097 (4)
O2	0.0440 (17)	0.075 (2)	0.068 (2)	−0.0179 (14)	0.0072 (15)	0.0119 (16)
O6	0.067 (2)	0.064 (2)	0.064 (2)	−0.0197 (16)	0.0253 (17)	−0.0072 (15)
O7	0.078 (2)	0.065 (2)	0.078 (2)	0.0016 (17)	0.0283 (19)	0.0181 (18)
N5	0.0447 (19)	0.0431 (18)	0.0466 (19)	0.0030 (14)	0.0189 (15)	−0.0066 (14)
C9	0.052 (3)	0.120 (5)	0.061 (3)	0.019 (3)	−0.004 (2)	−0.035 (3)
O8	0.062 (3)	0.045 (2)	0.085 (3)	0.000	−0.004 (2)	0.000

### Geometric parameters (Å, °)

**Table:**

Mn1—O1	2.176 (2)	C4—C5	1.491 (5)
Mn1—O1^i^	2.176 (2)	C6—C7	1.364 (6)
Mn1—N1^i^	2.258 (3)	C6—H6	0.9500
Mn1—N1	2.258 (3)	C7—C8	1.386 (5)
Mn1—N4^i^	2.249 (3)	C7—H7	0.9500
Mn1—N4	2.249 (3)	C8—H8	0.9500
O1—H1A	0.8400	Cl1—O5B	1.380 (11)
O1—H1B	0.8400	Cl1—O3B	1.387 (9)
N1—C4	1.332 (4)	Cl1—O4A	1.410 (6)
N1—C1	1.351 (4)	Cl1—O3A	1.413 (6)
N2—C4	1.326 (4)	Cl1—O2	1.420 (3)
N2—C3	1.348 (5)	Cl1—O4B	1.454 (10)
N3—C6	1.322 (5)	Cl1—O5A	1.521 (8)
N3—C5	1.337 (4)	O6—N5	1.199 (4)
N4—C8	1.337 (4)	O7—N5	1.213 (4)
N4—C5	1.341 (4)	N5—C9	1.441 (5)
C1—C2	1.369 (5)	C9—H9A	0.9800
C1—H1	0.9500	C9—H9B	0.9800
C2—C3	1.372 (6)	C9—H9C	0.9800
C2—H2	0.9500	O8—H8A	0.8400
C3—H3	0.9500		
			
O1—Mn1—O1^i^	90.36 (14)	N3—C5—C4	118.5 (3)
O1—Mn1—N4^i^	161.83 (10)	N4—C5—C4	116.6 (3)
O1^i^—Mn1—N4^i^	90.14 (9)	N3—C6—C7	123.1 (3)
O1—Mn1—N4	90.14 (9)	N3—C6—H6	118.4
O1^i^—Mn1—N4	161.83 (10)	C7—C6—H6	118.5
N4^i^—Mn1—N4	94.98 (13)	C6—C7—C8	117.0 (4)
O1—Mn1—N1^i^	89.27 (9)	C6—C7—H7	121.5
O1^i^—Mn1—N1^i^	94.93 (9)	C8—C7—H7	121.5
N4^i^—Mn1—N1^i^	72.59 (10)	N4—C8—C7	121.2 (4)
N4—Mn1—N1^i^	103.25 (10)	N4—C8—H8	119.4
O1—Mn1—N1	94.93 (9)	C7—C8—H8	119.4
O1^i^—Mn1—N1	89.27 (9)	O5B—Cl1—O3B	110.9 (6)
N4^i^—Mn1—N1	103.25 (10)	O5B—Cl1—O4A	75.8 (5)
N4—Mn1—N1	72.59 (10)	O3B—Cl1—O4A	129.3 (5)
N1^i^—Mn1—N1	174.05 (14)	O5B—Cl1—O3A	130.9 (6)
Mn1—O1—H1A	118.8	O4A—Cl1—O3A	112.2 (4)
Mn1—O1—H1B	117.4	O5B—Cl1—O2	102.5 (5)
H1A—O1—H1B	115.3	O3B—Cl1—O2	110.6 (4)
C4—N1—C1	116.7 (3)	O4A—Cl1—O2	116.8 (3)
C4—N1—Mn1	116.8 (2)	O3A—Cl1—O2	113.9 (3)
C1—N1—Mn1	125.9 (2)	O5B—Cl1—O4B	109.4 (6)
C4—N2—C3	115.0 (3)	O3B—Cl1—O4B	119.3 (5)
C6—N3—C5	116.6 (3)	O3A—Cl1—O4B	94.0 (5)
C8—N4—C5	117.1 (3)	O2—Cl1—O4B	102.5 (5)
C8—N4—Mn1	126.2 (2)	O3B—Cl1—O5A	81.1 (5)
C5—N4—Mn1	116.4 (2)	O4A—Cl1—O5A	100.5 (4)
N1—C1—C2	120.9 (3)	O3A—Cl1—O5A	104.3 (4)
N1—C1—H1	119.6	O2—Cl1—O5A	107.3 (3)
C2—C1—H1	119.6	O4B—Cl1—O5A	134.3 (5)
C1—C2—C3	117.6 (4)	O6—N5—O7	122.0 (4)
C1—C2—H2	121.2	O6—N5—C9	118.6 (4)
C3—C2—H2	121.2	O7—N5—C9	119.4 (4)
N2—C3—C2	122.9 (3)	N5—C9—H9A	109.5
N2—C3—H3	118.6	N5—C9—H9B	109.5
C2—C3—H3	118.6	H9A—C9—H9B	109.5
N2—C4—N1	126.8 (3)	N5—C9—H9C	109.5
N2—C4—C5	117.6 (3)	H9A—C9—H9C	109.5
N1—C4—C5	115.7 (3)	H9B—C9—H9C	109.5
N3—C5—N4	124.9 (3)		
			
O1—Mn1—N1—C4	76.5 (2)	C1—C2—C3—N2	1.5 (6)
O1^i^—Mn1—N1—C4	166.8 (2)	C3—N2—C4—N1	−3.2 (5)
N4^i^—Mn1—N1—C4	−103.2 (2)	C3—N2—C4—C5	176.5 (3)
N4—Mn1—N1—C4	−12.1 (2)	C1—N1—C4—N2	3.0 (5)
O1—Mn1—N1—C1	−94.8 (3)	Mn1—N1—C4—N2	−169.1 (3)
O1^i^—Mn1—N1—C1	−4.5 (3)	C1—N1—C4—C5	−176.6 (3)
N4^i^—Mn1—N1—C1	85.5 (3)	Mn1—N1—C4—C5	11.2 (3)
N4—Mn1—N1—C1	176.6 (3)	C6—N3—C5—N4	2.8 (5)
O1—Mn1—N4—C8	89.8 (3)	C6—N3—C5—C4	−176.3 (3)
O1^i^—Mn1—N4—C8	−178.6 (3)	C8—N4—C5—N3	−3.1 (5)
N4^i^—Mn1—N4—C8	−72.7 (3)	Mn1—N4—C5—N3	171.0 (2)
N1^i^—Mn1—N4—C8	0.6 (3)	C8—N4—C5—C4	176.0 (3)
N1—Mn1—N4—C8	−175.0 (3)	Mn1—N4—C5—C4	−9.8 (3)
O1—Mn1—N4—C5	−83.7 (2)	N2—C4—C5—N3	−1.5 (4)
O1^i^—Mn1—N4—C5	7.9 (5)	N1—C4—C5—N3	178.2 (3)
N4^i^—Mn1—N4—C5	113.8 (2)	N2—C4—C5—N4	179.4 (3)
N1^i^—Mn1—N4—C5	−173.0 (2)	N1—C4—C5—N4	−0.9 (4)
N1—Mn1—N4—C5	11.4 (2)	C5—N3—C6—C7	−0.3 (6)
C4—N1—C1—C2	−0.4 (5)	N3—C6—C7—C8	−1.6 (6)
Mn1—N1—C1—C2	170.9 (3)	C5—N4—C8—C7	0.9 (5)
N1—C1—C2—C3	−1.7 (6)	Mn1—N4—C8—C7	−172.6 (3)
C4—N2—C3—C2	0.7 (5)	C6—C7—C8—N4	1.3 (6)

Symmetry codes: (i) −*x*, *y*, −*z*+1/2.

### Hydrogen-bond geometry (Å, °)

**Table:**

*D*—H···*A*	*D*—H	H···*A*	*D*···*A*	*D*—H···*A*
O1—H1A···N2^ii^	0.84	2.48	3.286 (4)	162.
O1—H1A···N3^ii^	0.84	2.29	2.879 (4)	128.
O1—H1B···O8^iii^	0.84	1.97	2.757 (4)	156.
O8—H8A···O2^iv^	0.84	1.95	2.792 (4)	175.
C3—H3···O6^v^	0.95	2.54	3.346 (5)	143.
C6—H6···O3A^vi^	0.95	2.58	3.239 (7)	127.
C8—H8···O6^vii^	0.95	2.59	3.175 (5)	120.
C9—H9C···O5A	0.98	2.52	3.388 (9)	148.

Symmetry codes: (ii) −*x*, −*y*+2, −*z*+1; (iii) *x*, *y*+1, *z*; (iv) −*x*+1/2, *y*+1/2, −*z*+1/2; (v) −*x*+1/2, −*y*+3/2, −*z*+1; (vi) *x*−1/2, −*y*+1/2, *z*+1/2; (vii) *x*−1/2, *y*+1/2, *z*.

## References

[bb1] Bruker (2000). *SADABS*, *SMART* and *SAINT* Bruker AXS Inc., Madison, Wisconsin, USA.

[bb2] Farrugia, L. J. (1997). *J. Appl. Cryst.* **30**, 565.

[bb3] Ha, K. (2011). *Acta Cryst.* E**67**, m656–m657. 10.1107/S1600536811015388 PMC3120370 21754569

[bb4] Hong, D. M., Wei, H. H., Gan, L. L., Lee, G. H. & Wang, Y. (1996). *Polyhedron*, **15**, 2335–2340.

[bb5] Sheldrick, G. M. (2008). *Acta Cryst.* A**64**, 112–122. 10.1107/S0108767307043930 18156677

[bb6] Spek, A. L. (2009). *Acta Cryst.* D**65**, 148–155. 10.1107/S090744490804362X PMC2631630 19171970

